# Global scene layout modulates contextual learning in change detection

**DOI:** 10.3389/fpsyg.2014.00089

**Published:** 2014-02-10

**Authors:** Markus Conci, Hermann J. Müller

**Affiliations:** Allgemeine und Experimentelle Psychologie, Department Psychologie, Ludwig-Maximilians-Universität MünchenMünchen, Germany

**Keywords:** local/global processing, visual attention, change blindness, change detection, natural scenes, contextual learning

## Abstract

Change in the visual scene often goes unnoticed – a phenomenon referred to as “change blindness.” This study examined whether the hierarchical structure, i.e., the global–local layout of a scene can influence performance in a one-shot change detection paradigm. To this end, natural scenes of a laid breakfast table were presented, and observers were asked to locate the onset of a new local object. Importantly, the global structure of the scene was manipulated by varying the relations among objects in the scene layouts. The very same items were either presented as global-congruent (typical) layouts or as global-incongruent (random) arrangements. Change blindness was less severe for congruent than for incongruent displays, and this congruency benefit increased with the duration of the experiment. These findings show that global layouts are learned, supporting detection of local changes with enhanced efficiency. However, performance was not affected by scene congruency in a subsequent control experiment that required observers to localize a static discontinuity (i.e., an object that was missing from the repeated layouts). Our results thus show that learning of the global layout is particularly linked to the local objects. Taken together, our results reveal an effect of “global precedence” in natural scenes. We suggest that relational properties within the hierarchy of a natural scene are governed, in particular, by global image analysis, reducing change blindness for local objects through scene learning.

## INTRODUCTION

**FIGURE 1:**
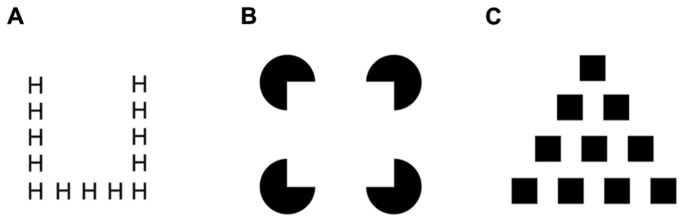
**Examples of hierarchical stimulus configurations with global and local levels of representation: (A) Navon letter, **(B)** Kanizsa square, and **(C)** hierarchical shape**.

Complex natural environments require the visual system to provide structure to the visual input, integrating fragmentary parts into coherent objects that are segregated from the background and other objects. The available, individuated objects of a given natural scene are usually represented hierarchically, with multiple levels of representation ranging from more global to more local instantiations. For example, a forest has trees and the trees in turn have leaves, illustrating a hierarchical relationship between parts and wholes at different levels of perceptual resolution. Similar hierarchical relations have also been demonstrated for a variety of composite figures that require global- and local-level structural elements to be integrated (see, e.g., Hübner and Volberg, 2005). For instance, the Navon letter (Navon, 1977) depicted in **Figure 1A** consists of an arrangement of the local letters “H,” which combine to form the global letter “U.” Similarly, Kanizsa figures (Kanizsa, 1976) comprise a local arrangement of circular inducers, which yield the impression of a salient, global square shape (**Figure 1B**). Comparable relationships can also emerge from other hierarchical shape stimuli, were, for instance, local squares combine to form a global triangle (**Figure 1C**; Kimchi and Palmer, 1982).

While object information can be accessed at various levels, attentional orienting by default exhibits “global precedence” (Navon, 1977). A number of studies demonstrated that visual search is based on global-level representations that comprise integrated objects, while fragmentary (local) object parts are not accessible with comparable efficiency (Pomerantz et al., 1977; Donnelly et al., 1991; Rensink and Enns, 1995; Rauschenberger and Yantis, 2001; Conci et al., 2007a,b, 2011; Deco and Heinke, 2007). For instance, processes of target detection in visual search prioritize complete global shape representations in Kanizsa figures or Navon letters, while the corresponding local parts are more difficult to access (e.g., Conci et al., 2007b; Deco and Heinke, 2007). Taken together, these finding suggests that the “forest comes before the trees” (Navon, 1977).

Priority of global over local levels of representation may also be related to a phenomenon referred to as “change blindness,” which demonstrates a striking inability to detect changes that occur in the visual ambient array (Simons and Levin, 1997; Rensink, 2002 for reviews). For example, in the flicker paradigm (Rensink et al., 1997), an original image and a slightly modified image of a natural scene are presented in rapid alternation with a blank screen interposed between them. Given this sequence of images, observers are usually relatively poor at identifying the object that changes across both views of the same scene. For instance, observers quite frequently do not detect a change within the first alternation of the images. In many cases, they even fail to detect a change after 1 min of continuous image flicker, or when the eyes fixate relatively close to the changed location (O’Regan et al., 2000). By contrast, when the two images are presented without a blank, changes are easily detected, due to the transients that mark the changed object. Given this pattern of results, it appears that the internal representation of the outer world is rather sparse, providing only the rough “gist” of a scene that is carried over from one instance to the next (e.g., Simons and Chabris, 1999). The sparse gist of the available information after a change may in turn be attributable to global precedence. That is, global, “forest”-like scene properties are registered more readily, whereas local changes (e.g., to trees and leaves) go relatively unnoticed. In this view, scene memory is primarily reliant upon global image analysis, thus preventing detection of local change.

Given this weak ability to register changes across views of a scene, orienting attention to the changed (local) objects is a key requirement for actually detecting what has changed (Rensink et al., 1997; Simons and Levin, 1997; see also Simons and Chabris, 1999). One possibility to increase the detectability of a change is by means of providing top-down information, for instance, giving observers a verbal cue (Rensink et al., 1997). In addition, change detection can also be improved to a certain extent by the degree to which a changed object is accompanied by salient bottom-up signals, such as luminance, color, or motion changes (Cole et al., 2004; Arrington et al., 2006; Cole and Liversedge, 2006). Compared to such changes within an existing object, the sudden appearance of a new object (or the sudden disappearance of an object) is most effective for reducing change blindness (Mondy and Coltheart, 2000; Scholl, 2000; Cole et al., 2004).

Besides bottom-up and top-down factors enhancing target detection, relational scene properties that potentially alter the basic structure of a scene have also been found to modulate change blindness. For example, when varying relational grouping cues (Jiang et al., 2004) or the assignment of figure and ground (Landman et al., 2004) between two successive images, change detection performance is severely impaired. By contrast, a reduction of change blindness is obtained when a given perceptual change alters the scene gist (Sampanes et al., 2008). Consequently, these results show that the relational properties of a scene (i.e., the spatial relations of objects among each other) critically influence change detection performance.

Invariant relational properties of a scene not only support detection of a change at a given instance, but can also influence long-term adaptive processes. For example, when a given target object is repeatedly paired within a consistent surround of contextual items, change detection performance is improved (Jiang and Song, 2005). Thus, repetition of the spatial contextual layout increases the detectability of a changed item. Jiang and Song(2005; see also Chun and Jiang, 1998) interpreted their findings in terms of an (implicit) mechanism that automatically associates a given target object with the statistically invariant relations given by the scene (i.e., the repeated context). Consequently, when observers are presented with an arrangement of items, associations will be formed between the target object and its surrounding context of neighboring objects, thus facilitating detection of the target on future occasions when it is presented within the same configuration. Of note, contextual learning also manifests in naturalistic environments, with scene memory linking target locations to invariant configurations, such as the spatially distributed arrangements of objects in indoor scenes (Brockmole et al., 2006). Accordingly, contextual memory may provide ecologically valid cues in predicting potential target locations, and learning the co-occurrence relations of objects in the environment can help guide behavior.

**FIGURE 2:**
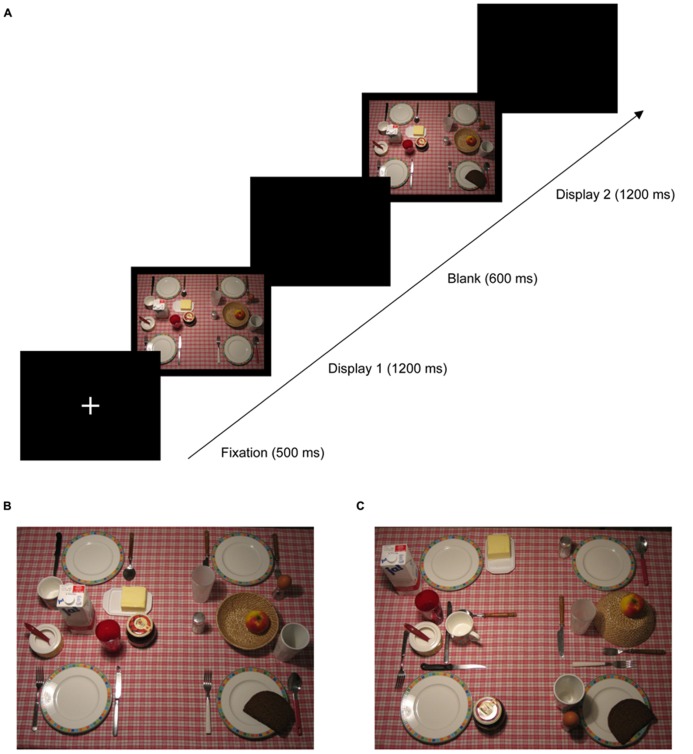
**(A)** Schematic trial sequence in Experiment 1: each trial started with a fixation cross presented for 500 ms, followed by two search displays presented for 1200 ms each and separated by a blank display presented for 600 ms. Participants were required to indicate whether a change occurred on the left or the right side of the screen. Panels **(B,C)** depict examples of global-congruent and -incongruent display layouts, respectively.

The aim of the present study was to investigate in further detail how relational structures of a scene can be acquired through contextual learning. Previous studies (described above) have shown that change blindness can be reduced when the change is associated with the global scene gist. In the current study, we investigated how *learning* of such global hierarchical structures in a natural scene can influence change detection. Observers were required to detect a local-object change within a natural (breakfast table) scene that presented global arrangements with either a congruent or an incongruent global scene structure (see **Figure 2**). Importantly, the same scene was presented repeatedly throughout the experiment to enable learning of the invariant object layout. Thus, we examined whether the structure provided by the global layout would affect learning of contextual regularities and, in turn, what effect learning would have on the detection of (local) changes.

## EXPERIMENT 1

Experiment 1 was designed to test whether learning of *hierarchical structure* in a natural scene context can influence change blindness. To this end, observers were presented with a sequence of two search displays (i.e., a “one-shot” procedure; e.g., Cole et al., 2004; Jiang and Song, 2005; Cole and Liversedge, 2006) that consisted of photographs depicting a laid breakfast table (see **Figure 2**). As illustrated in **Figure 2A**, on each trial, one item was added to the second search display, and observers were required to indicate the side on which the change had occurred (left vs. right half of the screen). Importantly, there were two types of displays: for the global-congruent display layout, all items on the breakfast table were placed such that the global structure of the table resembled a prototypical, meaningful arrangement (**Figure 2B**). In contrast, for the global-incongruent display layout, the very same local items were presented at comparable eccentricities and orientations, but the arrangement was pseudo-random such that the typical, “meaningful” structure of the scene was missing (**Figure 2C**). Thus, in congruent displays, the relational structure among the objects provided a coherent global organization of the scene – so that to-be-detected changes of local objects were accompanied by an “intact” global configuration. The identical local objects were also available in the incongruent condition – however, the coherent global scene structure was lacking. Consequently, comparisons of both types of layout would indicate whether the analysis of global image properties influences change detection performance over and above the (salient) signal provided by the onset of the local target object itself. Moreover, we examined whether detectability of the local target varies over the duration of the experiment, to assess whether the concomitant learning of contextual regularities is modulated by the congruency of the global scene structure.

### MATERIALS AND METHODS

#### Participants

Sixteen observers (eight male; mean age = 27 years; normal or corrected-to-normal visual acuity) participated in the experiment.

#### Ethics statement

The present study, including the consent procedure, was approved by the ethics board of the LMU Munich Psychology Department and conducted according to the principles of the Declaration of Helsinki. Participants received information about the study and their rights and gave informed verbal consent.

#### Apparatus and stimuli

The experiment was controlled by an IBM-PC compatible computer using Matlab routines and Psychophysics Toolbox extensions (Brainard, 1997; Pelli, 1997). Stimuli were presented on a 17-inch monitor (at a frame rate of 85 Hz). On each trial, two successive search displays were presented for 1200 ms, with an offset of 600 ms in-between the display presentations (see **Figure 2A** for an example trial sequence). Search displays were color pictures of a laid breakfast table (1024 × 768 pixels), subtending approximately 34° × 28° of visual angle, and photographed from a bird’s eye view. Each picture consisted of 26 objects, placed on the background of a tablecloth with a red and white checkerboard pattern. All objects were typical breakfast items (four plates, two glasses, two cups, three spoons, three forks, three knifes, milk, butter, jam, bread, butter, egg, salt, sugar, apple, bread basket) that were distributed equally across the left and right halves of the screen. Displays on a given trial could be from either the global-congruent or the global-incongruent display layout condition. Congruent displays (see **Figure 2B**) presented all local objects in a prototypical global arrangement, with the breakfast items placed at “standard” locations (e.g., forks and knifes were presented next to the plate, butter and jam were located in the central region of the table). By contrast, with incongruent layouts (see **Figure 2C**), the very same local items were presented at comparable eccentricities and orientations; however, the global organization of the breakfast table was lacking its typical arrangement (e.g., forks and knifes were presented at the center of the table, whereas butter and jam were located next to the plate).

On a given trial, the two sequential displays were either both from the global-congruent or both from the global-incongruent condition (**Figure 2A**). In the first display, one object was missing on the breakfast table, whereas the second display always consisted of the complete layout of 26 objects (**Figures 2B,C**). Thus, there was always an onset of a new object in the second display. There were 20 objects that served as onset objects, while six of the 26 objects (four plates, sugar, basket; distributed equally across both halves of the screen) always remained stationary across the display sequence. Subjects were instructed to indicate, as accurately as possible, whether a change (i.e., the onset of a new object in the second display) had occurred on the left or the right side of the screen.

#### Trial sequence

Each trial started with the presentation of a central fixation cross for 500 ms. Next, the first display (with one object missing) was presented for 1200 ms. Following a blank screen of 600 ms, the second (complete) display was presented for another 1200 ms. Finally, a blank screen was presented during which the participants were instructed to give a non-speeded response via mouse keys. Participants’ task was to indicate the side of the screen (left or right) on which a change (i.e., an object onset) had occurred. In case of an erroneous response, feedback was provided by an alerting sign (“–”) presented for 1000 ms at the center of the screen. Trials were separated from each other by an interval of 1000 ms. **Figure 2A** illustrates a typical trial sequence with an object onset (slice of bread) occurring in the bottom right quadrant of the screen.

#### Design and procedure

A two-factors within-subjects design was used. The independent variables were display type and block. Display type had two levels: global-congruent and global-incongruent. For global-congruent displays, all items were arranged such that the image of the laid table had a prototypical layout. In contrast, for the global-incongruent trials, the same items were arranged at comparable eccentricities and orientations, though with a pseudo-random stimulus configuration such that all items were clearly visible but lacked the global structure given in the congruent display condition. The second variable, block, simply divided the experiment into five consecutive bins, to permit examining for possible learning effects over the course of the experiment.

At the beginning of the experiment, participants completed one block of 40 practice trials to become familiarized with the task. All subsequent experimental blocks contained 20 global-congruent and 20 global-incongruent trials, presented in randomized order, such that in each bock, all 40 different target objects were presented once. There were five blocks in the experiment, yielding 200 experimental trials in total.

### RESULTS AND DISCUSSION

Accuracy of detecting the changes across display sequences was relatively high, with an average of 76% of correct responses across all conditions.

**FIGURE 3:**
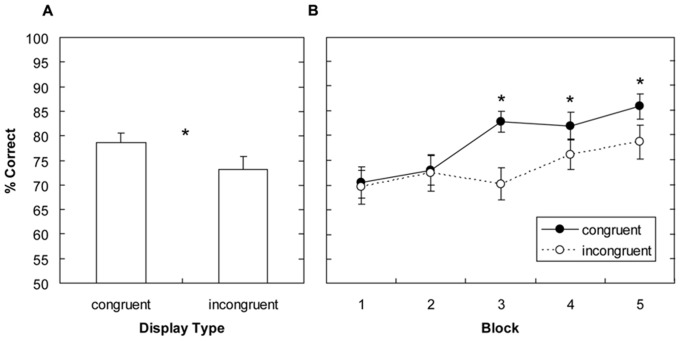
**Experiment 1: mean percentage of correct responses for global-congruent and -incongruent display layouts (A), and corresponding average response accuracies plotted as a function of block **(B)**.** Significant differences revealed by pairwise comparisons are indicated by an asterisk. Error bars denote the standard error of the mean.

The mean correct responses were analyzed by means of a two-way repeated-measures analysis of variance (ANOVA) with main terms for display type (global-congruent, global-incongruent) and block (1–5). This analysis revealed significant main effects for display type, *F*_(1,15)_ = 14.76, *p* < 0.003, and block, *F*_(4,60)_ = 7.63, *p* < 0.001. The main effect of display type was due change detection being more accurate, by 5.4%, for global-congruent as compared to global-incongruent display layouts (**Figure 3A**). The main effect of block indicated that responses became, in general, more accurate as the experiment progressed (70%, 73%, 76%, 79%, and 82% for blocks 1–5). Importantly, the display type by block interaction was also significant, *F*_(4,60)_ = 2.67, *p* < 0.05, owing to the fact that the advantage for congruent display types was not evident right from the beginning, but became reliable only as the experiment progressed: As can be seen from **Figure 3B**, there were no significant differences in blocks 1 and 2 (mean difference: 0.8%, all *p*s > 0.7); but from block 3 onwards, changes were detected more accurately with global-congruent compared to global-incongruent display layouts (mean difference: 8.5%, all *p*s < 0.03). Thus, this analysis shows that global congruent scene layouts attenuated change blindness. In addition, accuracy increased in general with repeated exposure of the changed items across blocks, but this performance gain was particularly pronounced for global-congruent display layouts. This suggests that learning of the global-congruent displays gradually facilitated detection of the object onsets.

In a subsequent step, an additional analysis was performed to further investigate what was actually learned in the display layouts: it could be the case that the increase in accuracy across blocks was due to the repeated exposure to the scene layouts, which might have facilitated object detection through learning the relational scene structure (e.g., Chun and Jiang, 1998). Alternatively, it could be that on each trial, observers registered and assigned an “inhibitory tag” (e.g., Klein and MacInnes, 1999) to the location of the changed target object; as a result, on subsequent trials, already registered target locations would then be inhibited – effectively biasing attention towards locations that so far (within a given block) had not contained a target. Thus, on this account, observers learn to inhibit locations at which a change had occurred on previous trials, permitting them to anticipate the locations of the upcoming targets with increasing validity across the trials within a block (as the number of alternative locations decreases). To examine whether this form of inhibitory tagging might explain the current results, blocks 3–5 (which showed evidence for a benefit for global-congruent layouts) were each separated into two halves. Evidence for inhibition of already detected changes should then result in an increase in performance for the second half of each block, as the number of potential target locations decreases with each additional trial. A repeated-measures ANOVA performed on the collapsed data from blocks 3–5 with main terms for display type (global-congruent, global-incongruent) and block half (first, second) revealed the display type effect to be significant, *F*_(1,15)_ = 37.52, *p* <0.001, essentially mirroring the above difference between global-congruent and -incongruent displays. However, there was no main or interaction effect that included block half (all *p*s >0.2), at variance with inhibitory location tagging across trials in the current experiment (accuracies were 79% and 78% for the first and second block half, respectively). Note that this null-effect also rules out accounts on which internal search “templates” for already detected target objects, rather than their scene locations, are inhibitorily tagged (e.g., along the lines of Houghton and Tipper, 1994). Thus, by default, the performance benefit for congruent displays is likely attributable to contextual learning of the global scene layout (rather than a particular “anticipation” strategy).

## EXPERIMENT 2

Having established that global scene structure reduces change blindness after several exposures, in particular for congruent layouts, Experiment 2 was designed to further examine possible causes of this effect. One possibility is that detection of the change in global-congruent layouts is facilitated by scene memory; that is, learning of the scene context may lead to more efficient encoding and, as a result, enhanced detection of the changed (target) object. Alternatively, however, familiarity with a given layout after a few trials might also facilitate localization of the target just by inspecting the pre-change display. Thus, for instance, by searching for the object that is missing from pre-change display, observers might be able to detect an inconsistency in the scene without actually comparing the pre- and post-change displays. That is, observers might come to use – over repeated encounters – their acquired knowledge of what the search layout should look like, giving rise to an effect of familiarity on the detection of the missing object without contextual learning coming playing a role. Thus, on this alternative account, the results of Experiment 1 are attributable to a relatively general effect of contextual familiarity on search, rather than a specific influence of contextual learning on the localization of a target. It should be noted that this explanation would not necessarily require inhibitory tagging within a block of trials (see above), as scene inconsistencies might be detected without registration of previous targets.

Experiment 2 was performed to decide between these two possible explanations. Observers were presented with a single image of a scene (identical to the first displays in Experiment 1) and asked to indicate the side on which an object was missing within the scene. Thus, if observers detect the missing object in repeated layouts on the basis of familiarity, then scene congruency should modulate performance in a way comparable to Experiment 1. Conversely, no influence of scene congruency should be evident if contextual learning is specifically associated with detection of a target object.

### MATERIALS AND METHODS

Apparatus, stimuli, design, and procedure were identical to Experiment 1, except that in Experiment 2, observers were presented with a single search display only. That is, on each trial, observers were presented with a fixation cross (500 ms), followed by a search display that was identical to the first display in Experiment 1 (i.e., one object was missing in each display). Observers’ task was to indicate the side of the screen on which one object was missing (left vs. right). Search displays remained on screen until a response button was pressed. Sixteen observers (seven male; mean age = 29 years; normal or corrected-to-normal visual acuity) participated in the experiment. All other details were identical to Experiment 1.

### RESULTS AND DISCUSSION

Overall, the missing objects in the displays were correctly “localized” in 69% of the trials averaged across all conditions; that is, response accuracy tended to be somewhat (by 7%) lower overall compared to Experiment 1, *t*_(30)_ = 1.89, *p* = 0.07 – though performance was still comfortably above chance level (50%).

**FIGURE 4:**
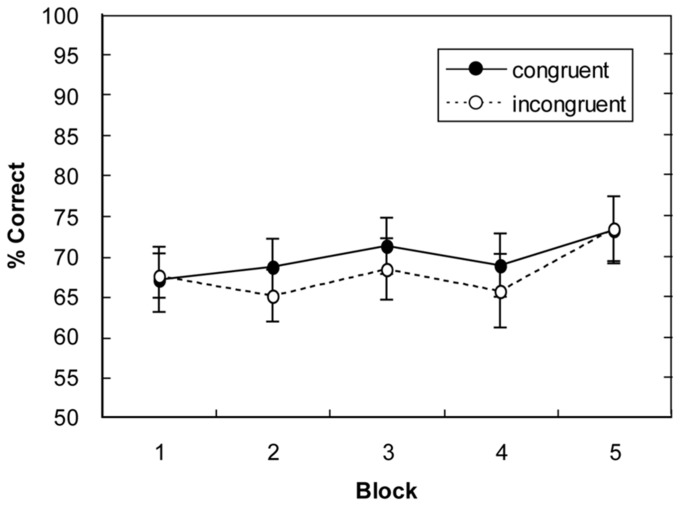
**Mean percentage of correct responses for global-congruent and -incongruent display layouts as a function of block in Experiment 2.** Error bars indicate the standard error of the mean.

A repeated-measures ANOVA of the percentages of correct responses, with main terms for display type (global-congruent, global-incongruent) and block (1–5), revealed only a marginally significant main effect of block, *F*_(4,60)_ = 2.04, *p* = 0.09, but no main effect of display type (*p* = 0.21) and no display type × block interaction (*p* = 0.86). As can be seen from **Figure 4**, there was some increase in response accuracy over the course of the experiment (from 67% in block 1 to 73% in block 5). However, in contrast to Experiment 1, there was no influence of the type of display on performance accuracy (responses were, on average, 0.9% more accurate for global-congruent than for global-incongruent layouts; all *p*s > 0.17).

In sum, Experiment 2 revealed no evidence for a modulation of change detection as a function of scene congruency. Thus, in a task that required the detection of a static discontinuity (i.e., a missing object in a previously encountered scene layout), observers were unable to exploit the global contextual regularities associated with a given display. Responses were just 0.9% more accurate to global congruent than to global incongruent item arrangements, which contrasts with a clear advantage for congruent layouts in Experiment 1, exhibiting an increase in accuracy by ~5% across the entire experiment (or of 8.5% in blocks 3–5). Thus, the benefit of global scene congruency seems to be specifically related to the detection of the target object’s appearance by means of a pre- vs. post-change comparison (as in Experiment 1). Despite the lack of a global scene congruency benefit in Experiment 2, observers were able to detect the missing objects above chance in congruent as well as incongruent layouts. This suggests that observers can – to some extent – localize a missing object on the basis of familiarity, while an additional benefit may derive specifically for global congruent displays via the learning of target–context associations (as indicated by the results of Experiment 1).

## GENERAL DISCUSSION

The present study examined whether the global layout, provided by the relational structure of a natural scene, can influence change blindness. The results of Experiment 1 indicated that change detection in global-congruent displays is less impaired than detection within global-incongruent layouts. This congruency benefit was, however, not evident immediately from the beginning of the experiment; rather, it emerged only after observers had experienced several repetitions of the scene. Given this delayed onset, perceptual (i.e., structural) differences between the displays cannot account for this effect, as these should have been effective right from the start. Thus, the fact that the difference between display layouts became manifest only after several repetitions can be taken to indicate that the scene context was learned. Additional analyses suggested that this benefit derived from learning of the (contextual) scene layout, rather than from inhibiting all detected (previous) changes throughout a block of trials. Thus, more information about the scene layout and potential changes of objects (rather than changes at a single target location; see Chun and Jiang, 1998) was accumulated over several repetitions of global-congruent displays, permitting changes to be registered with greater accuracy.

While there was an influence of contextual learning on change detection in Experiment 1, no comparable effect materialized with the altered task introduced in Experiment 2: search for an inconsistency in the scene (i.e., search for a “missing” object) was not modulated by the global structure given by the arrangement of the items. This outcome shows that the influence of the global scene layout in Experiment 1 was critically related to the localization of the changed local object (involving a comparison of the pre- and post-change displays), rather than to more efficient processing of familiar layouts *per se*. Restated, observers could not learn to use the familiar scene layouts to detect the missing objects. If anything, then familiarity facilitated overall performance for all types of layout as the experiment progressed.

Several perceptual and strategic factors have so far been shown to influence the detectability of changes. For instance, top-down information (Rensink et al., 1997) or the type of bottom-up stimulus (Mondy and Coltheart, 2000; Scholl, 2000; Cole et al., 2004; Arrington et al., 2006; Cole and Liversedge, 2006) can provide target-relevant information modulating change blindness. However, besides factors that enhance processing of the target object itself, the relations between objects in a scene can alter the detectability of a changed object (Jiang et al., 2004; Landman et al., 2004). Conversely, objects that modulate the gist of a scene (Sampanes et al., 2008) or objects that are particularly relevant for understanding the meaning of a scene (Kelley et al., 2003) can reduce change blindness. In general agreement with these studies, the current study demonstrates that the relational global structure of a scene layout modulates detection of a local object onset.

A number of studies that employed compound stimuli in visual search suggest that global-level configurations receive attentional priority over local object components (Navon, 1977; Pomerantz et al., 1977; Donnelly et al., 1991; Rensink and Enns, 1995; Rauschenberger and Yantis, 2001; Conci et al., 2007a,b, 2011; Deco and Heinke, 2007). A potential explanation for these findings of “global precedence” suggests that the visual system first extracts the rather crude global “wholes” of a scene (via feedforward processes) at preattentive stages, while local details become available only subsequently and require additional (recurrent) processing (e.g., Hochstein and Ahissar, 2002; Conci et al., 2009). Such a differentiation between global and local processing routines may, in turn, not only explain results from visual search studies with compound stimuli (as described above), but could also be linked to evidence from natural scene perception, which shows that the rough gist of a scene becomes available at brief presentation durations (Thorpe et al., 1996) without the engagement of attentional resources (Li et al., 2002), and probably relying on rather crude, low-spatial frequency channels that encode in particular global scene components (De Cesarei and Loftus, 2011). In this regard, the present results complement these findings and demonstrate that global scene analysis may also be mediated via learning: preserved global structure can facilitate learning of a given display layout, which in turn reduces change blindness.

In fact, our results suggest a rather specific function of memory in viewing natural scene contexts: repeating targets (across blocks) improved performance, and this benefit was particularly pronounced for targets embedded within global-congruent contextual layouts. Thus, potential targets were learned in relation to the scene context, in line with the view that coherent global scene layout specifically modulates learned associations between repeated targets and contexts (Conci and Müller, 2012, see also Brockmole et al., 2006; Hollingworth, 2009). Conversely, there was no benefit of searching repeatedly for different objects. For instance, within a given block, there was no evidence of inhibitory tagging of the locations at which a target had been detected on previous trials. Also, there was no benefit when searching for a “missing” object, that is, a “discontinuity” in a familiar scene layout (Experiment 2). This pattern is in line with recent work by Võ and Wolfe (2012), which revealed no effects of “familiarity” when searching for different target objects within repeated scenes, but clear benefits when the target objects themselves were repeated. This suggests that global scene layouts are associated with recurring targets via scene learning. It should be noted, however, that no such effect of learning is found when varying target locations in an “artificial” letter search task (Wolfe et al., 2000; Zellin et al., 2011), which indicates that coherent, global scene information is needed to effectively integrate multiple target associations within a single context.

Unlike simple, “artificial” display layouts, natural scenes typically contain meaningful objects that are associated with specific affordances that can be linked to actions (Gibson, 1979). For example, the knife on the breakfast table “affords” the action of picking it up. Accordingly, a natural scene context might thus provide stronger predictive cues for guiding behavior, as objects might be directly relevant for a given action. In this regard, the use of artificial search displays might impose a certain limit on contextual learning, as a given meaningless layout is likely to be less effective in yielding reliable memory traces. By contrast, natural scenes provide rich contexts that allow for more reliable predictions, and these appear to be modulated in particular by the available global structure.

In general, coherent scene layouts have been shown to influence performance in a variety of tasks. For example, a loaf of bread is identified more accurately in the context of a kitchen than in that of a front yard surround (Palmer, 1975). In addition, when a scene is scrambled (i.e., cut into sections which are then randomly rearranged) such that the relations among the objects are disturbed, identification of a target object is impaired relative to a coherent scene (Biederman, 1972). Also, the context of a scene can be used to guide attention to likely target locations (Neider and Zelinsky, 2006), yielding more efficient search when a target location is constrained by scene context. In agreement with these findings, global-incongruent display layouts in the current study were processed less effectively and led to more change blindness than global-congruent displays.

The gradually evolving benefit for global-congruent scene layouts evidences an influence of contextual learning on target detection. In general, invariant spatial contexts can influence visual search (Chun and Jiang, 1998) and change detection (Jiang and Song, 2005). Moreover, context-based learning has been shown to be highly sensitive to processes of object grouping and segmentation (Conci and von Mühlenen, 2009, 2011; Conci et al., 2013), suggesting that relational properties determine how a context is learned. Complementary results were obtained here for “higher-order” relations in a real-world (breakfast) scene. Scene-congruent relations among objects were learned more readily, reducing change blindness. Thus, this finding extends earlier work in showing that global layout can influence the encoding of context, modulating performance in change detection experiments. More broadly, the present study demonstrates that the global gist of a scene contributes to the registration of local changes in dynamic environments – illustrating that global precedence can link “forests” to “trees” for efficient orienting in natural scenes.

## Conflict of Interest Statement

The authors declare that the research was conducted in the absence of any commercial or financial relationships that could be construed as a potential conflict of interest.
